# Anal extraskeletal osteosarcoma in a man: a case report and review of the literature

**DOI:** 10.1186/s13256-020-02365-1

**Published:** 2020-04-20

**Authors:** Ramin Saadaat, Jamshid Abdul-Ghafar, Nasir Ud Din, Ahmed Maseh Haidary

**Affiliations:** 1 Department of Pathology and Laboratory Medicine, French Medical Institute for Mothers and Children (FMIC), Kabul, Afghanistan; 2 Department of Pathology and Laboratory Medicine, Aga Khan University Hospital (AKU-H), Karachi, Pakistan

**Keywords:** Anus, Extraskeletal, Osteosarcoma, Older age, Poorer prognosis

## Abstract

**Background:**

Osteosarcoma is a common malignancy of bone that usually occurs in individuals in the age range of 0–24 years. Extraskeletal osteosarcoma is a rare tumor presentation which originates in non-bony tissues. Extraskeletal osteosarcoma comprises 2–5% of all osteosarcomas and less than 1% of all soft tissue sarcomas. As compared to bone-derived osteosarcoma, extraskeletal osteosarcoma occurs in older age groups. Extraskeletal osteosarcoma has a poorer prognosis than bone osteosarcoma. To the best of our knowledge, this is the first case of extraskeletal osteosarcoma in the anal region.

**Case presentation:**

A 70-year-old Hazara man presented to a private hospital with the chief complaints of constipation, bloody defecation, and pain during defecation of 1.5 months’ duration. His past history was unremarkable. A digital rectal examination showed a solid growth in the middle part of his anus. A colonoscopic examination was done and showed a solid mass in his anal region. A computed tomography scan revealed an irregular mural thickening in the anal canal with heterogeneous enhancement. The maximum length of the involved segment was measured to be 4.5 cm. No suspicious lesions were noted in other organs. An abdominoperineal resection was performed on our patient. A 22 cm in length resected segment of his colon, consisting of the lower sigmoid, rectum, and anus was sent to us for histopathological examination. Gross examination revealed a polypoid dark-gray tumor measuring 5 × 3 × 2 cm. The cut section revealed gray and white appearance with firm-to-hard consistency and foci of ossification. Microscopic examination revealed normal anorectal mucosa and a spindle cell malignant neoplasm with osteoid formations. No evidence of epithelial carcinoma was noted. Immunohistochemical stains were positive for stabilin-2 and negative for cytokeratin, which confirmed the diagnosis of osteosarcoma.

**Conclusion:**

Extraskeletal osteosarcoma of the colon is rare and presence of the tumor in the rectum and anal region is extremely rare. Radiology, colonoscopy, and histopathology with immunostaining are required for the diagnosis. The accurate diagnosis of extraskeletal osteosarcoma is important as it has a different regimen of treatment with poorer prognosis compared to primary osteosarcoma of the bone.

## Introduction

Osteosarcomas derive from primitive mesenchymal cells characterized by the presence of malignant mesenchymal cells that produce osteoid. Osteosarcoma most commonly originates from bone and rarely from soft tissue with the age range of 0–24 years [1]. The average incidence rate of osteosarcoma among young individuals is 4.3 per million in men and 3.4 per million in women, with a male-to-female ratio of 1.4:1 [2].

Osteosarcoma may develop *de novo* or in combination with well-known precursors such as Paget’s disease, radiation injury, bone infarcts, osteomyelitis, and certain clinical syndromes [3]. Extraskeletal osteosarcoma (ESOS) is a rare malignant mesenchymal-derived tumor. ESOS originates in non-skeletal soft tissues. ESOSs are much rarer than skeletal osteosarcomas [4]. ESOSs comprise 2–5% of all osteosarcomas and less than 1% of all soft tissue sarcomas [5]. ESOS was first described in the thyroid and gallbladder by Wilson in 1941 [6]. ESOS can occur in any part of the body, with common sites of involvement being lower extremities, upper extremities, retroperitoneal region, and buttocks [7].

ESOSs have also been reported in some unusual locations, such as larynx, kidneys, esophagus, small bowel, liver, heart, urinary bladder, salivary gland, and breast [8]. A review of the literature showed that the occurrence of primary ESOS in the gastrointestinal tract (GIT) was very rare. To date, only two cases have been reported in the esophagus [9, 10], one in the transverse colon [11], one in the rectum [12], and one in the jejunum [13] (Table 1).

**Table 1:** Previoous reported cases of primary ESOS in gasatrointestinal tract

Author (Year) Reference number	Anatomic site	Age (year)	Gender
McIntyre *et al.* (1982) [10]	Esophagus	70	M
Erra *et al*. (2010) [9]	Esophagus	74	M
Nojima *et al*. (1986) [13]	Jejunum	23	M
Shimazu *et al*. (2001) [11]	Transverse colon	53	F
Iannaci *et al*. (2013) [12]	Rectum	81	F
Current case	Anal canal	70	M

*F* female, *M* male

The exact etiology of this tumor is unknown, although 5–10% of cases occur after radiotherapy, and preceding trauma is reported in 12–13% of cases. Distant metastases occur most frequently in lungs, lymph nodes, and bone [14].

Surgical resection is the standard treatment. ESOSs are considered poorly responsive to chemotherapy. Ahmad *et al.* [15] reported that only 19% respond with complete remission to doxorubicin-based chemotherapy and 13% to cisplatin-based chemotherapy. However, because of the perceived similarities to skeletal osteosarcoma, the role of chemotherapy is debated.

Here we report the first case of primary ESOS of the anal area, which is an extremely rare location for this tumor.

## Case presentation

A 70-year-old retired Hazara man from a rural area of the country presented to a private hospital with the main complaints of constipation, bloody defecation, and pain during defecation of 1.5 months’ duration. He did not narrate history of any remarkable disease or symptoms in the past. No family history was present. A laboratory examination showed low hemoglobin (Hb) of 9.3 m/dl with normal range of blood urea nitrogen (BUN), creatinine, alkaline phosphatase, and bilirubin. A digital rectal examination was performed and showed solid growth in the middle part of his anal canal. Colonoscopic findings showed a polypoid solid mass in lower anorectal junction. A computed tomography (CT) scan revealed an irregular mural thickening in the anal canal with heterogeneous enhancement which had a maximum length of 4.5 cm. No suspected lesions were noted in his liver and lungs and there were no abnormalities noted in the bony skeleton on physical examination.

**Fig. 1:**
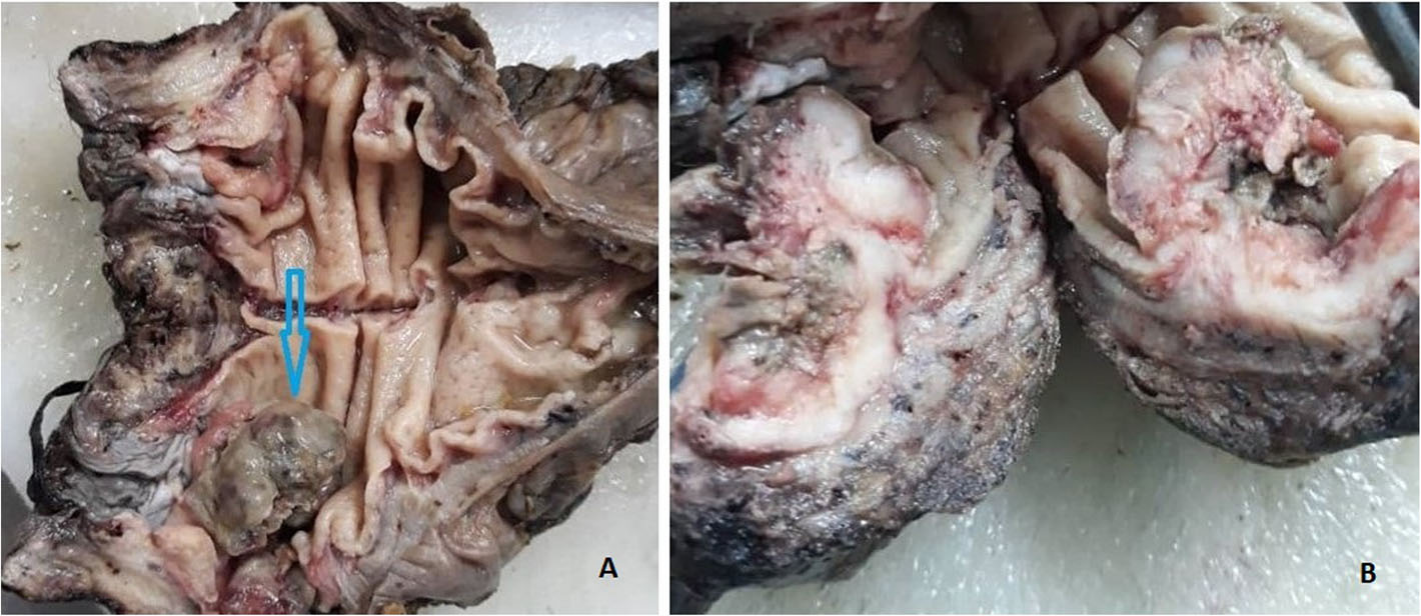
Gross images of the specimen. **a** Gray and dark exophytic tumor located in anal area (*arrow*). **b** Sectioning of the tumor across the adventitia showing white and pink appearance with firm and few hard bony cut surfaces

An abdominoperineal resection (APR) was performed and the surgically resected specimen was sent for histopathologic examination to the Department of Pathology and Laboratory Medicine, French Medical Institute for Mothers and children (FMIC). A 22 cm in length resected segment of the colon, consisting of lower sigmoid, rectum, and anus was received in formalin fixation. After opening through antimesenteric border, it revealed a polypoid dark-gray tumor in the anal area, measuring 5 × 3 × 2 cm in its dimensions (Fig. 1a). The tumor was located approximately 2 cm from anal resection margin. The cut section of the mass had gray and white appearance with firm-to-hard consistency and foci of ossifications (Fig. 1b).

**Fig. 2:**
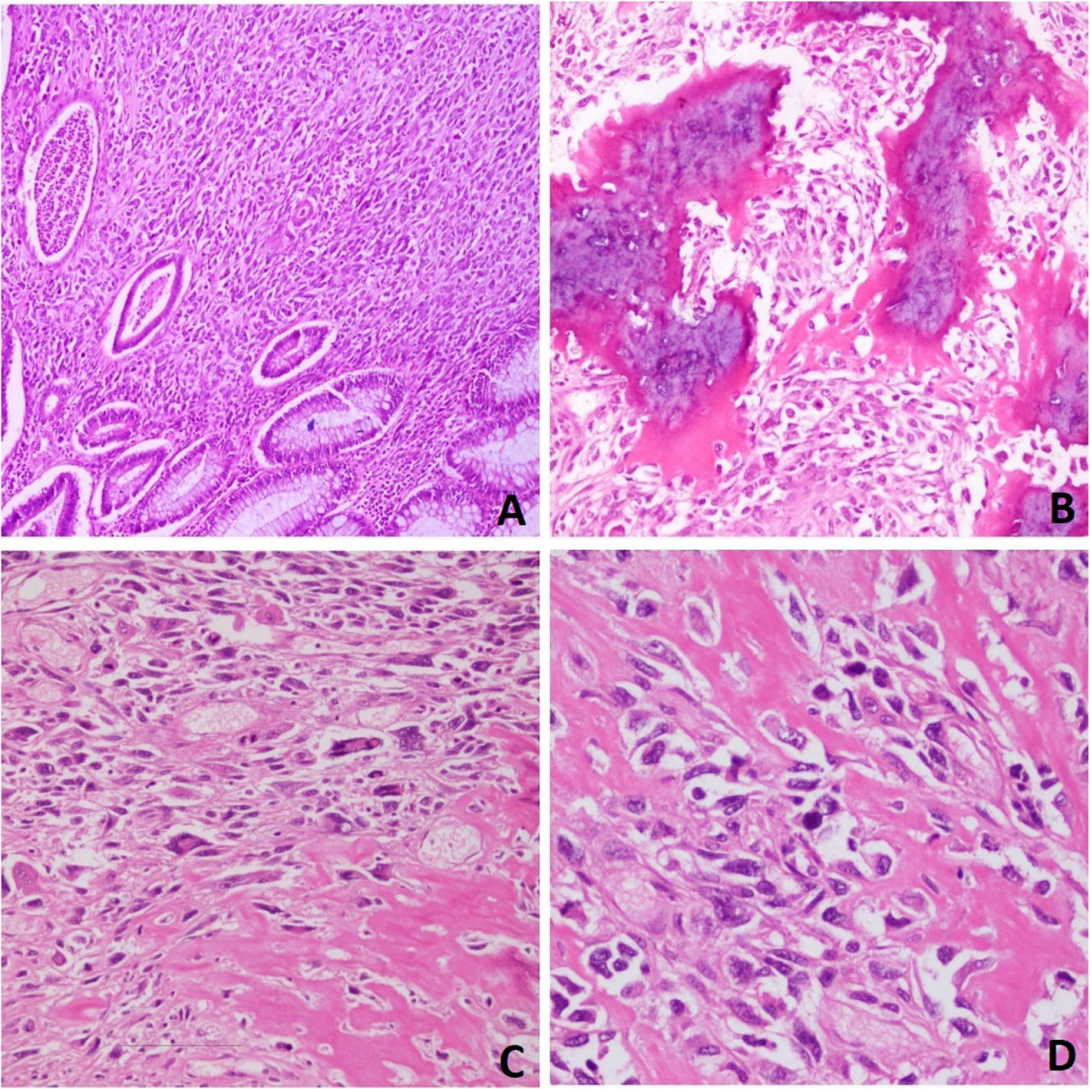
Microscopic findings of the tumor stained with hematoxylin and eosin. **a** Normal glands of the anal mucosa with a spindle cell neoplasm in the lamina propria and submucosa (magnification, × 100). **b** and **d** Osteoid formation between spindle cells (magnification, × 200). **c** Pleomorphic and atypical spindle cell neoplasm with osteoid formation (magnification, × 200 and magnification, x 400)

**Fig. 3:**
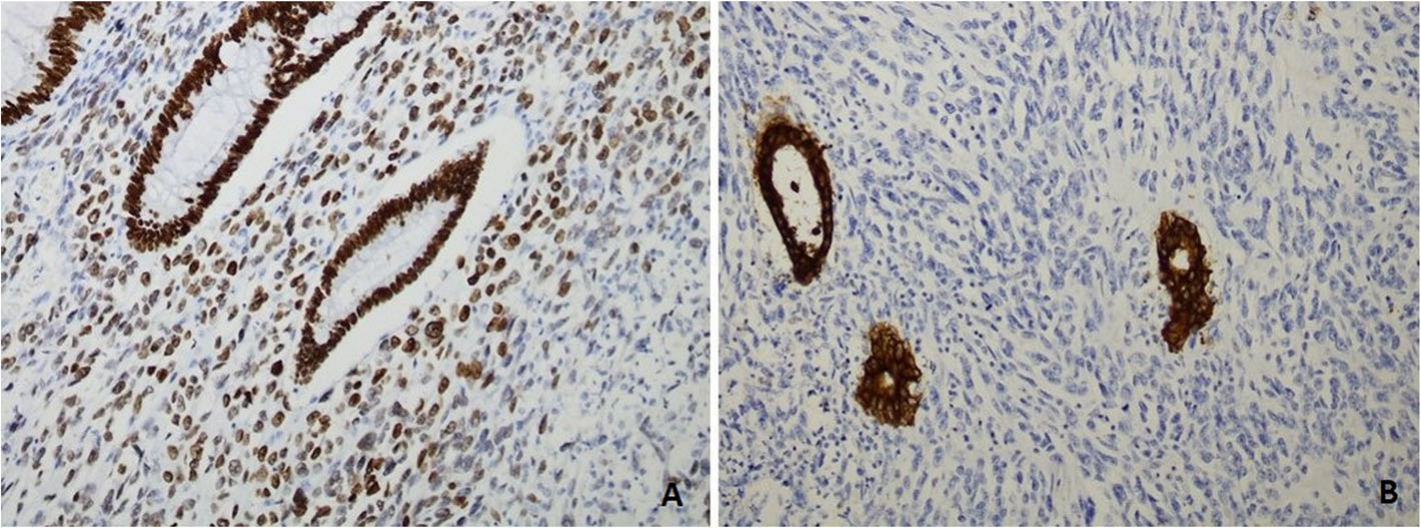
Immunohistochemical stains. **a** Tumor cells were positive for stabilin-2 and **b** negative for cytokeratin AE1

On microscopic examination, it revealed normal anorectal mucosa lined by benign columnar epithelium. The lamina propria and submucosa showed sheets of spindle cell neoplasm arranged in a haphazard pattern. The neoplastic cells were large pleomorphic with hyperchromatic nuclei and prominent nucleoli. Many osteoid formations and immature bony tissues were noted between the tumor cells (Fig. 2a–d). No evidence of epithelial carcinoma was noted in the multiple sections that were examined. Immunohistochemical (IHC) stains showed that tumor cells were positive for stabilin-2 (STAB2) and negative for pan-cytokeratin (CK AE1), which confirmed the diagnosis of ESOS (Fig. 3a, b).

## Discussion

ESOS is a rare malignant neoplasm with unknown etiology. Two theories exist regarding the pathogenesis of ESOS: The first theory states that osteosarcoma develops in mesoblastic tissue residues of embryonic origin. According to the second theory, it has been suggested that interstitial fibroblasts in smooth muscle when exposed to external or internal stimulation (such as trauma or inflammation) undergo osteoblastic or chondroblastic metaplasia, which ultimately evolves into osteosarcoma [16].

The most common location for primary ESOS is the soft tissues of the lower extremities, predominantly thigh (46%), followed by the upper extremities (20%) and the retroperitoneum (17%), but it can occur in any part of the body [17]. Cases of ESOS have been reported in the gallbladder [18], liver [19], ovary [20], pineal region [21], penis [22], esophagus [9], breast [23], and head of the pancreas [24]. Most cases of ESOSs were reported in male individuals [14].

Primary ESOS in the colorectal area is extremely rare. To date, only two cases of primary osteosarcoma of the colon and rectum have been reported. Both cases were reported in female patients of 53 [11] and 81 [12] years of age. The present case is primary ESOS involving the anal area, which is the first case of ESOS in this location. The age of the patient in the current case was 70-year-old at the time of diagnosis.

By definition, ESOS is located in extraskeletal tissue without primary involvement or connection to the bone or periosteum [4]. Three diagnostic criteria for ESOS have been proposed by Lee *et al.* [16]: (i) the mass should arise from soft tissue and not be connected to bone or skeleton, (ii) the morphology of the tumor must be the same as osteosarcoma, and (iii) the tumor must produce osteoid or cartilaginous matrix. Our case fulfills all the above criteria, as the tumor arose from the wall of the anal canal, which had no connection to bone, and had the histologic morphology of osteosarcoma with osteoid production interspersed between tumor cells.

Many histologic patterns are available. In the osteoblastic type of ESOS, bone matrix is abundant. In the fibroblastic subtype, spindle cells with herringbone or storiform pattern characterize the lesion. In the chondroid variant, a predominance of malignant cartilage tissue is seen, and the cells usually have large nuclei with irregular contours. In some cases, collagen is abundant in extracellular matrix and electron-dense crystals of hydroxyapatite are present in areas of bone deposition [25].

Metastatic osteosarcoma should be ruled out during the diagnosis of primary ESOS. Osteosarcomas most commonly metastasize to the lungs, bones, pleura, and heart and less commonly to the liver, lymph nodes, and brain, but rarely metastasize to GIT [26]. The most common metastatic tumor to GIT is melanoma followed by ovarian, bladder, breast, and lung tumors [27]. In our case, a CT scan of our patient revealed no mass or lesion in bone and other organs of his body. Moreover, patients with bone osteosarcoma are younger than patients with primary ESOS [2]; therefore, the younger age of patient with metastatic osteosarcoma can also be a differential factor. The age of the patient in the present case was 70-year-old, which is not the usual age for primary bone osteosarcoma.

Primary ESOS should also be differentiated from radiotherapy-induced osteosarcoma. The most common soft tissue sarcomas which arise after radiotherapy for breast and other cancers are angiosarcoma and osteosarcoma [28]. The pathogenesis is unknown but the occurrence of osteosarcoma after radiation can be predisposed by various factors, such as dosage of radiation, associated chemotherapy, age of the patient, and genetic susceptibility to oncogenesis [29]. The incidence rate of radiotherapy-associated ESOS is 3.8–10% of all ESOSs [16] and 13% of all radiation-induced sarcomas [30]. Most cases of osteosarcoma after radiation arise from skeletal structures, but radiation-induced osteosarcoma can be extraskeletal [31]. In 1948, Cahan *et al.* [32] established four criteria for the diagnosis of radiation-induced osteosarcoma, which are still valid: (i) history of radiation therapy, (ii) sarcoma arising within the radiation field after the radiation has been given, (iii) long latency period, and (iv) histologic confirmation of the sarcoma. The patient in the current case had no history of radiotherapy, so we can exclude radiotherapy-induced ESOS.

In addition, ESOS should be differentiated from morphologically similar tumors. One of the tumors which should be in a differential diagnosis is bone and cartilage formation in malignant fibrous histiocytoma (MFH). MFH is a spindle cell tumor and it rarely contains bony and cartilaginous components [33]. The differential points of MFH from ESOS are: bony and cartilaginous elements in MFH are metaplastic in appearance, similar to periosteal fracture callus; and located only as focal areas, mostly in the periphery of the tumor and in relation to the tumor pseudo-capsule. Differentiation between ESOS and MFH is important as both have different regimes of treatment and prognosis, with MFH having a relatively better prognosis than ESOS [33]. Thus, the histologic findings of our case were consistent with osteosarcoma rather than metaplastic bone in other mesenchymal neoplasms.

Careful attention is necessary to exclude the diagnosis of metaplastic carcinoma which includes both epithelial and mesenchymal components [34]. In the current case, an absence of epithelial components excluded the diagnosis of a metaplastic carcinoma.

## Conclusion

Primary ESOS of the anorectal region is extremely rare. Radiology, colonoscopy, and histopathology with IHC stains are required to confirm the diagnosis. The accurate diagnosis of ESOS is important because its poorer prognosis and different regimen of treatment compare to the primary bone osteosarcoma.

## Data Availability

All data generated or analyzed during this study are included in this published article.

## Abbreviations

APR: Abdominoperineal resection
BUN: Blood urea nitrogen
CK: Cytokeratin
CT: Computed tomography
ESOS: Extraskeletal osteosarcoma
FMIC: French Medical Institute for Mothers and Children
GIT: Gastrointestinal tract
Hb: Hemoglobin
IHC: Immunohistochemical
MFH: Malignant fibrous histiocytoma
STAB2: Stabilin-2
